# The evaluation of health, disability and aged care-sector engagement with resources designed to support optimisation of the allied health assistant workforce: a qualitative study

**DOI:** 10.1186/s12913-024-11253-z

**Published:** 2024-07-26

**Authors:** Lucy Whelan, Sharon McLean, Alexandra Edwards, Jessica Huglin, Melanie K. Farlie

**Affiliations:** 1 Allied Health Workforce Innovation, Strategy, Education & Research (WISER) Unit, Monash Health, 400 Warrigal Road, Cheltenham, VIC 3192 Australia; 2 Faculty of Medicine, Nursing and Health Sciences, Monash University, 27 Rainforest Walk, Clayton, VIC 3168 Australia

**Keywords:** Allied health, Assistant, Support, Workforce, Career, Delegation

## Abstract

**Background:**

Allied health assistants (AHAs) support allied health professionals (AHPs) to meet workforce demands in modern healthcare systems. In an Australian context historically, AHAs have been sub-optimally utilised. Prior research has identified that AHAs and AHPs working in health, disability and aged care sectors, and the Vocational Education and Training (VET) industry, may benefit from access to resources to support the optimisation of the AHA workforce. As a part of a Victorian department of health funded project, several resources were developed in line with workforce recommendations for use in each of the above sectors. Recommendations and resources covered the broad areas of pre-employment training, workforce planning and governance, consumer-centred therapy and supports, recruitment and induction and workplace training and development. This study aimed to evaluate the engagement with these newly designed resources to support optimisation of AHAs in the Victorian context.

**Methods:**

Semi-structured interviews were conducted to evaluate engagement with resources, from the perspective of AHAs, AHPs and allied health leaders (AHLs) in the health, aged care or disability sectors, and educators and managers of allied health assistance training. Thematic analysis was conducted using team-based framework analysis.

**Results:**

Thematic framework analysis of the interview data identified four themes; *Why participants accessed the resources; How participants engaged with the resources; What (if any) changes in practice occurred as a result of engaging with the resources in a participant’s local context, How did participants envision the resources being utilised for AHA workforce optimisation in the future*. Responses were mapped to the AHA workforce career pathway at the career preparation, career development and career trajectory tiers.

**Conclusions:**

Appetite for AHA workforce development and optimal utilisation is evident across Victoria, Australia. Readily accessible resources that inform AHA role and scope of practice, delegation practice, or improve the ability for an AHA to state their own development needs, were identified as useful by participants. The potential for these resources to assist in the optimal utilisation and development of AHA workforces across the career continuum differs according to the role, sector and geographical location of the resource user. Further study is needed to investigate the transferability of these resources to national and global contexts.

**Supplementary Information:**

The online version contains supplementary material available at 10.1186/s12913-024-11253-z.

## Background

Allied health assistants (AHAs) are an established support workforce across Australian health, disability and aged care settings [1]. AHAs assist allied health professionals (AHPs) such as physiotherapists, podiatrists, speech pathologists, social workers, dietitians, and occupational therapists to provide therapeutic care [1]. AHAs perform a range of delegated therapeutic tasks under the direction of the AHP. This enables the AHP to focus on the work that requires their expertise and provide care to a greater number of people. An AHA’s duties may include providing individual therapy sessions, running a therapy group, setting up equipment and some administrative duties. In Victoria, the second most populous state in Australia, AHAs work in a variety of settings including health, aged care and disability. With an increasing demand for AHAs in the context of AHP workforce shortages [2], recent evidence has focused on the optimal utilisation of AHAs [3–6].

It is well understood across sectors, that optimising the AHA workforce is a plausible solution to addressing some of the issues faced by the health, disability and aged-care sectors. Despite multiple existing guidelines and frameworks, evidence suggests that the AHA workforce is not currently optimised in their development, nor their utilisation [3–6]. Pre-employment training of AHAs is variable, scope of practice is ambiguous, delegation practice is not well understood by AHPs, and targeted workplace training for this workforce is limited [3]. Barriers to and enablers of optimal utilisation of the AHA workforce in the Victorian context, have been explored previously [3]. The factors influencing AHA utilisation were identified in this previous study as belonging to the following categories; pre-employment training, system factors, individual factors and workplace factors. Furthermore, these factors were found to operate across three Allied health assistant career phases: Career preparation, Career Development and Career Trajectory [3]. The study findings reported by Huglin et al. (2021) [3], led to the development of resources, auspiced by the Victorian Department of Health [7], aimed at optimising the utilisation of AHAs in Victorian health, aged care and disability sectors. These online resources, which target a number of the factors influencing AHA utilisation [3], were released online in March 2022, and as of November 2023, had over 25,000 webpage visits and more than 7,500 individual resource downloads [7]. The fourteen resources represent five main categories; pre-employment training, workforce planning and governance, consumer-centred therapy and supports, recruitment and induction and workplace training and development, and include templates such as position descriptions and delegation tools. Additional file 1 lists these resources.

## Methods

### Aims

The aim of this study was to evaluate how AHAs, AHPs and Allied health leaders (AHLs) working in health, disability and aged care sectors and the Vocational Education and Training (VET) industry, engaged with these new resources, and to ascertain if participants perceived the resources as likely to support the optimisation of the AHA workforce in their respective contexts.

### Study design and research team

This qualitative study was conducted from an interpretivist perspective. The research team sought to understand and construct meaning from participant reports of their engagement with online resources. This study used a team-based framework analysis approach [8] to describe participants’ engagement with a suite of online resources intended to support the optimisation of the AHA workforce, and then mapped the findings to an AHA Career Pathway previously reported by Huglin et al. (2021) [3]. This study builds on prior research describing the barriers and enablers to optimal utilisation of the AHA workforce in the Victorian context, by exploring how resources may support optimisation.

Research team members participated in a reflexivity exercise as described by Barry et al. (1999) [9], at the outset of this study, to establish a shared understanding of individual team member perspectives related to the project. Team members represented many years of allied health professional and assistant clinical practice, education and training, with varied levels of operational, governance, policy, leadership, and qualitative research experience. The research team met regularly throughout the project to review data collection, analysis and interpretation as a collective. Reporting of this study follows the Consolidated Criteria for Reporting of Qualitative Research (COREQ) [10]. Ethical approval was granted by the Monash Health Human Research Ethics Committee (RES-20-0000-356 L / ERM 64,899).

### Participants and recruitment method

Participants were recruited if they completed an optional expression of interest form when accessing the online resources. The existence of the online resources was communicated by email and newsletters distributed to existing contact lists associated with a Victorian AHA workforce project and via social media. Expressions of interest were collected between March and June of 2022. To access the resources, users were also asked to provide demographic information including their work role and sector (Health, Disability, Aged Care, Vocational Education and Training or other), their location (metropolitan, regional or outside Victoria) and the resources they intended to download.

Purposive sampling using quotas based on resource user demographics [11] was used to invite people who had expressed interest to participate in an interview. No repeat interviews were conducted. This sampling method was used to ensure participants represented AHAs and non-AHAs, as well as metropolitan and regional settings, and health, disability, aged care and education sectors. Eligible participants had to have downloaded at least one resource and be currently employed in the Victorian health, disability, aged care or VET sector, as either an AHA, AHP, AHL or an educator or manager of allied health assistance training. Individuals were excluded if they could not access a video conferencing platform to participate.

**Fig. 1:**
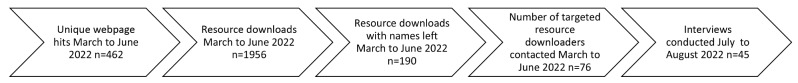
Interview candidate recruitment process

Figure 1 illustrates the process by which participants were recruited.

One thousand, nine hundred and fifty-six resources were downloaded between March 2022 and June 2022, of which one hundred and ninety individuals expressed interest in interview participation. Based on the purposive sampling grid, seventy-six individuals were contacted and invited to participate in a sixty-minute video interview, at a time convenient to them, within a four-week interviewing period. Table 1 depicts the demographic data associated with each participant. Additional File 2 depicts the demographic data of all downloaders between March and June 2022. Additional file 3 depicts intended downloads and actual downloads.

**Table 1:** Interview participant demographic data

Role/Sector	Disability	Health	VET	Other	Aged Care	Grand Total
AHA	3	6		1		10
AHL	7	6		2	4	19
**AHP**	**4**	**3**		**2**	**1**	**10**
**VET**			**6**			**6**
**Grand Total**	**14**	**15**	**6**	**5**	**5**	**45**

### Sources of data and data collection

A semi-structured interview guide (Additional file 4) was used to explore participant perspectives on how the online resources they engaged with, may support optimisation of the AHA workforce .The main topic areas in the interview guide focused on: why the participant had engaged with the resource/s being discussed, how they had used the resource/s, what changes (if any) had been required to the resources for the participant’s context, and any plans for ongoing use of resources. Interview recordings were transcribed verbatim by an external transcription service and anonymised prior to analysis. Structured post-interview debriefing notes were also completed by the interviewers after each interview and discussed with the research team to inform analysis, and identify when no new information was being garnered from interviews. At that stage the team came to a joint decision that sufficient information power had been achieved and the interview stage was finalised [12].

### Data analysis

Data analysis commenced from the first interview. Data was analysed using a framework approach to thematic analysis as described by Ritchie et al. (1999) [8]. Analysis steps included familiarisation, building a thematic framework, indexing, description and mapping. Four researchers (JH, MKF, SM, LW) became familiar with transcripts for two interviews each (eight in total) for the initial coding round. Domains of the interview guide (why, what, how and future) were used as an organising framework for the analysis. Data was deductively coded into these four categories, and then inductively coded to build the thematic framework. This was repeated for three rounds to achieve a stable thematic framework.

Once the thematic framework was devised (Additional file 5), one researcher (JH) indexed the entire dataset, with all members of the research team meeting regularly to discuss any additions or adjustments to the thematic framework, during the indexing stage. Once the dataset was fully indexed against the finalised thematic framework, one researcher (AE) mapped the dataset to the AHA Career Pathway elements described in Huglin et al. (2021) [3] (Additional file 6). All members of the research team continued to meet until the interpretation of the analysis was finalised. Qualitative analysis was managed in data analysis software program ATLAS.ti [13].

## Results

Seventy-six invitations were sent out. Seventeen participants declined to proceed with an interview and fourteen failed to attend the scheduled interview. Subsequently, forty-five semi-structured interviews were conducted from July 2022 to August 2022 by three researchers (LW, SM and JH), where eighty-one resources were discussed. While participants usually downloaded the suite of resources in their entirety, the resources most commonly selected by participants for discussion in the interviews were the AHA delegation tool (discussed in seventeen interviews), the grade two AHA position description template (discussed in sixteen interviews), the clinician checklist (discussed in twelve interviews) and the continuing professional development (CPD) log (discussed in six interviews).

The identified engagement with the resources, was represented by four themes: (1) why, (2) how, (3) what, and (4) future. Each theme contained multiple subthemes (Table 2). Main themes are presented first and then descriptions of how the themes were represented in the data when examined by sector, role and geography is presented. Lastly the mapping of the dataset to the AHA Career Pathway is presented.

**Table 2:** Code book structure of themes and sub-themes

**Theme 1: Why participants accessed the resources**	
1.01 To find a resource for your workplace/local need	
1.02 To find resources that had been informed by current evidence	
1.03 Planning for optimising utilisation of the AHA workforce	
1.04 To develop consistent pre-employment training	
1.05 To foster AHA work readiness	
1.06 To increase knowledge of AHA scope and role	
1.07 To address a gap in practice related to AHA workforce	
1.08 To confirm suitability of current practice	
1.09 Simplicity of resources	
1.10 To support governmental priorities	
**Theme 2: How participants engaged with the resources**	
2.01 By adapting resource to local needs	
2.02 By defining AHA scope and role	
2.03 To deliver AHA education	
2.04 By comparing/validating against existing tools	
**Theme 3: What (if any) changes in practice occurred as a result of engaging with the resources in a participant’s local context**	
3.01 Utilising AHAs optimally	
3.02 Creating new AHP and AHA teams/working relationships	
3.03 Improving understanding and definition of AHA roles	
3.04 Informing service delivery models	
**Theme 4: How did participants envision the resources being utilised for AHA workforce optimisation in the future**	
4.01 Creating positive attitudes towards AHA workforces/roles	
4.02 Ensuring equitable and accessible services	
4.03 Defining the AHA and AHP roles	
4.04 Creating consistency of resources training and workplaces	
4.05 Improving efficiency	
4.06 Future edits to the resources	
4.07 Increasing AHA workforces	
4.08 Developing AHA workforces	
4.09 Integration in pre-employment training	

### Theme 1: Why participants accessed the resources

Common sub-themes across sectors, as to why resources were accessed, were consistent with the enablers and barriers identified for the utilisation and development of the AHA workforce in previous research [3]. Participants from each sector, reported looking at the resources to cross-reference with their existing resources. Participants also described seeking out the resources to see if they could act as a starting point for AHA workforce development, where they had no existing resources; *“The position description was actually, probably, the thing that stood out for us, because we had only just moved into AHAs, and we had to write a position description. So that’s what I was actually looking for when I stumbled across the whole package, so that was incredibly helpful.” (AHP, Disability)*. Participants also reported looking at the resources to assess whether they were ready for use and easily adaptable to meet the needs of the AHA workforce in their local context.

All sectors identified the need to further clarify and understand the role of the AHA, as a key reason for downloading the resources; *“Broadly on delegation and actually having resources available, whether it’s yours [delegation tool resource] or looking towards our delegation training package being released, I think it helps with understanding and clarity around the benefits, the roles, the scope of allied health assistants, and gives people confidence to be able to move forward.” (AHL, Health).*

Participants from the aged care sector further described reasons for download of resources; to support the broader utilisation of AHAs, to address knowledge gaps and to clarify where unclear governance structures exist.

Participants from the VET sector reported seeking out the resources to address known barriers to AHA workforce optimisation such as inconsistent pre-training review to determine a candidate’s suitability to undertake the course and the variability in course content and delivery. Furthermore, participants from the VET and health sectors reported downloading resources for use in educational materials, when onboarding new staff and students on clinical placement.

### Theme 2: How participants engaged with the resources

Overall, participants across sectors expressed limited use of the resources, despite the interest in downloading the resources. Where the resources had been used in health and aged care, they had largely been used with only minor adaptations to conform with local documentation templates and branding; *“From a manager’s perspective, when you’ve got so many different types of interviews that you’re doing, particularly when they’re geared much more for clinicians, this was an invaluable document [the AHA interview guide]…that was really useful. It really did bring out that reflection piece from the person we were interviewing, and it was relevant to that person in terms of how they would approach AHA work and how they would approach communication with clients, and how they would document and all those things that are necessary for the AHA.” (AHL, Aged Care).*

Reported use within the disability sector included determination of AHA scope of practice and supporting documentation requirements. VET sector participants reported that resources were yet to be used, as any changes in that sector are contingent on first being accepted as part of any Registered Training Organisation (RTO) standards and accreditation requirements.

### Theme 3: What (if any) changes in practice occurred as a result of engaging with the resources in a participant’s local context

For participants working in aged-care, changes attributed to the use of the resources included a shift in thinking about the AHA role, using resources in education programs to support AHA clinical roles and support for improved service delivery (e.g. reduced waitlists and equity of consumer service access); *“And we’ve now been able to move one of our wonderful AHAs into a grade 3 role to help lead that development in the team, so these tools just help to create that structure and accountability, it’s wonderful.” (AHL, Aged Care).*

Participants from all sectors suggested that the resources helped them to identify future learning opportunities that may support AHA career progression and to inform clinical practice through professional development.

### Theme 4: How did participants envision the resources being utilised for AHA workforce optimisation in the future

The future vision for use of the resources expressed was similar across health, disability and aged care, in that the resources would aid consistent education to clarify the AHA role. It was further expressed that the resources would empower an AHA to speak to their role and their development needs, and further promote the value of the AHA role within the organization. In health specifically, the ability for an AHA to identify their own learning needs using these resources was articulated; *“I feel like it’s a tool [the clinician checklist] to clarify expectations, I think I just like the questions, the fact that they’re actually querying very specific topics, and then they are also outlining very clearly exactly what that means. I think that makes it very, very easy to follow for everyone, and the expectations are outlined really clearly there. I just found it, the language and the layout was quick to complete, and quick to go through, and really took you in your own mind exactly to the areas that were important for both parties.” (AHA, Health).*

VET sector participants reported that these resources would allow for an improved selection of appropriate students to undertake the course with the potential to better prepare students for the workplace, and allow for consistency in course delivery, by informing course content.

### Results described by sector

**Fig. 2:**
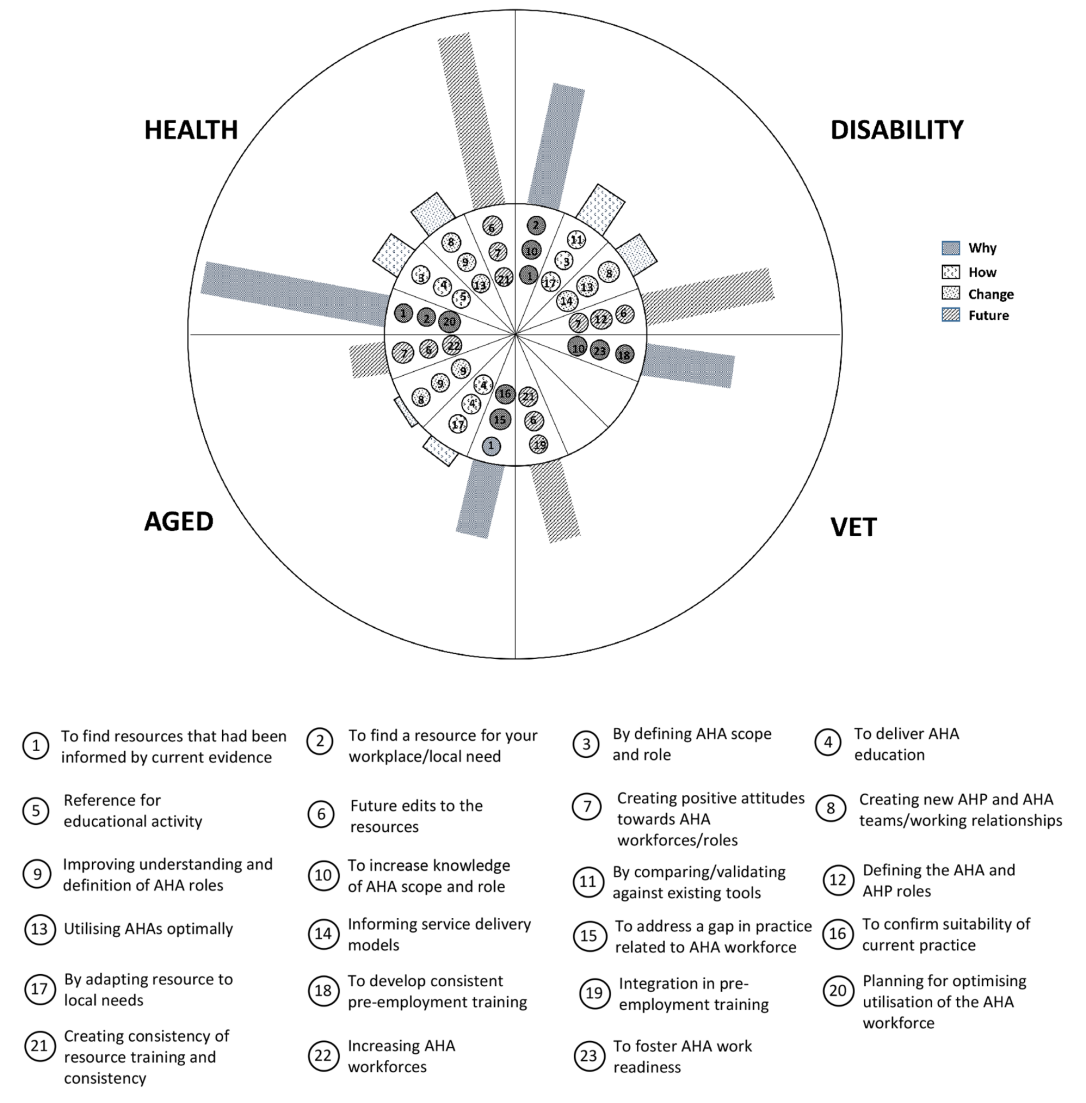
Visual representation of coding according to sector and category. The graphs represent the proportion of codes linking to the four themes of Change, Future, How and Why divided into each sector. The middle circle represents the three most commonly discussed topics within each theme. See legend for details

Participants from the health and the aged care sectors discussed *why* they had downloaded the resources more so, than *how* they had used them. However, they also reported more *future* intended use of the resources, suggesting that more time may be necessary to adopt the resources in each sector. Participants from the VET sector explained *why* they had downloaded the resources but reported limited actual use, again highlighting potential use in *future* planning, signaling a slower rate of adoption in this sector. The predominance of coding in the *why* (Theme 1) and *future* use (Theme 4) for all sectors may be related to the time frame between download and interview (as short as two weeks in some cases). Our data did indicate a quicker adoption of the resources in the disability sector as participants from this sector spoke the most about *how* they had used the resources and what they had changed as a result of using the resources. Figure 2 provides a graphical description of the four themes and the three most represented subthemes by sector.

### Results described by participant role

In health settings, AHA and AHP participants reported using resources as a cross-reference and directly adopting them for use. AHLs reported using the resources as a cross-reference, or to inform workplace preparation for AHAs: *“…that progress measurement tool… stood out for us is in terms of not losing sight of where we are at with where we’re progressing with our governance frameworks for Allied Health Assistants and even just the implementation of the use of them… the self-checking.”* (*AHL, Regional, Health*).

AHA and AHP participants from the disability sector reported using the resources to clarify scope and directly adopt, while AHLs reported using the resources to cross-reference existing resources or directly adopted them; *“we had to write a position description…I was just …just having a bit of a search what was around, and I stumbled across these resources. So I found it really helpful when we were trying to write the position description, and pulling it together” (AHP, Metropolitan, Disability).*

AHA, AHP and AHL participants from aged care settings all described using the resources to cross-reference existing resources where they existed and directly adopted them where possible. Aged care sector AHL participants spoke specifically about addressing delegation practice knowledge gaps with the resources, while AHAs spoke to the importance of the resources in clarifying the role of an AHA to consumers; *“I did a presentation this year as well through the Community Advisory Group here in the community. I felt like I’m sure they don’t even know what an Allied Health Assistant is. I used that tool [Consumer information] … in, my presentation. I used it a bit, … “What is an AHA compared to?” And they [said], “I had no idea, we thought you had four physios in your team.”* (*AHA, Regional, Aged Care*).

VET sector participants reported the resources would be useful in supporting pre-training review, identifying the appropriate course candidates, to cross-reference against existing education resources and to improve AHA student work readiness; *“…We would use these [position description] to update that part of the unit …when we speak about scope of practice, position description, role, everything that they do”* (*RTO representative, Metropolitan, VET*).

### Results described by geographical area

In this study, perspectives from metropolitan and regional health, aged care, disability and VET sectors were collected. Victorian regional services are generally smaller than their metropolitan counterparts, and potentially have an established AHA workforce with a less robust governance structure, whilst metropolitan services may be larger or networked such that governance structures are more robust, but potentially change is slower to adopt. Despite different patterns of download between metropolitan and regional participants (Additional file 7), minimal variation was noted between metropolitan and regional health participants. All participants reported similar uses for the resources in addressing local needs, using evidence-based resources and supporting optimisation of AHA workforces by defining scope of practice. Participants reported that awareness of the AHA role saw a positive shift in AHP and AHA working relationships. Both regional and metropolitan participants identified the resources as tools for changing attitudes; *“I think it [the CPD* l*og] makes you aware that that is available and that is a part of your development. And it creates that opportunity to view it and see, “Well, where do I want to go? What do I want to do?” and question yourself. Because I think sometimes in these roles, you do sit back and you’re just, “Well, that’s my role. That’s it.” But there isn’t. There’s so much more to it.”* (*AHA, Metropolitan, Health*). Participants planned to edit resources to suit individual needs and to improve attitudes regarding the AHA workforce.

Within the disability sector, metropolitan participants focused on utilisation of the current AHA workforce. Regional participants were focused on knowledge of AHA scope and role and confirming the suitability of their current practice with AHAs.

Participant perspectives from the aged care sector had some variation between metropolitan and regional participants. Metropolitan participants were focused on confirming the suitability of their current practice with AHAs and addressing any gaps in relation to the AHA workforce; *“the explanation about the [delegation] tool has been really useful, to be able to share that around that this is the expectation for effective delegation. And under the supervision delegation framework, it is – ‘You are ultimately responsible for your client and any risks and any issues need to be effectively communicated through to the AHA’, and that the AHA really understands what the expectations are. And that there’s timeframes put to that, and that they understand the urgency.”* (AHL, Metropolitan, Aged care). While participants in regional settings reported aiming to increase knowledge of the AHA role and sourcing evidence-informed resources to commence their AHA utilisation; *“I mean, at the moment I feel that we are just kind of putting a hand up, saying, “We want some people [AHAs] over here.” But it might be really good to do some targeted recruitment with some of this information [AHA interview guide and delegation tool] in our minds. It would even inform our advertisements, I think.”* (*AHL, Regional, Aged care*).

### Mapping of results to conceptual AHA Career pathway model

**Fig. 3:**
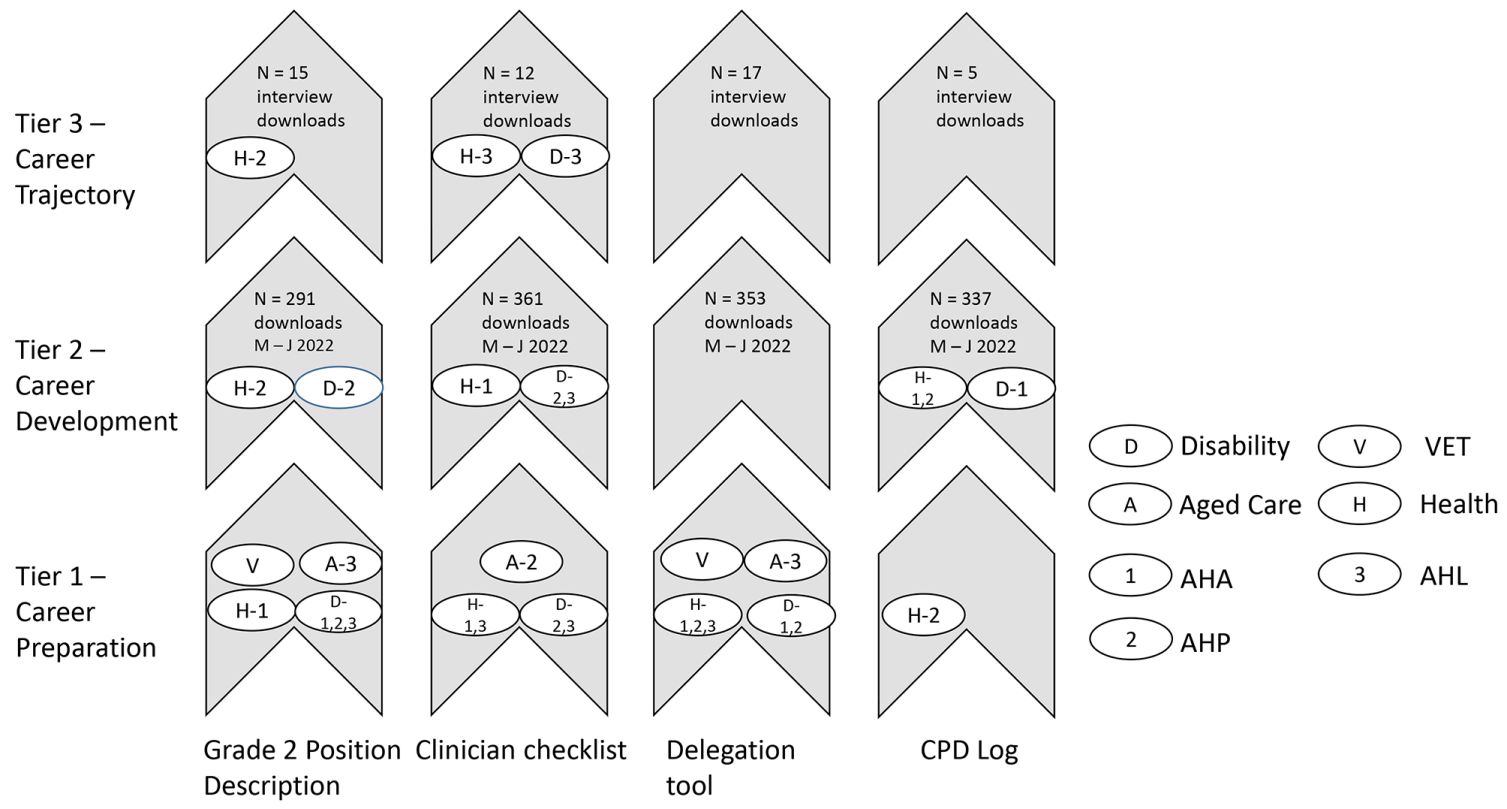
Utilisation of four resources mapped to career pathway tier (Tier 1, career preparation, Tier 2, career development and Tier 3, career trajectory) for an AHA within each sector. Note in Tier 2, N states the total number of downloads of the specific resource between March and June, Tier 3 states the number of downloads by participants only

The mapping of the data to an existing AHA career pathway model shows how engagement with resources related to the career pathway tiers of preparation, development and trajectory [3]. To map the resources with the most representation across the dataset, only the four most commonly discussed resources were mapped to the career pathway model. These resources were mapped against the career pathway tiers by participant group across each sector as shown in Fig. 3.

### Tier 1 – Career preparation

Participants from health, disability, aged-care and vocational training sectors reported utilising or plans to utilise the four mapped resources to set up workplace systems, culture and governance, to optimise the role of the AHA workforce in each sector. Participants from the health and disability sectors were utilising or planning to utilise the grade two position description in their AHA recruitment processes. Participants from health, disability and aged care were using or planning to use the clinician checklist to set up expectations and positive working relationships between AHPs and AHAs. The grade two position description and the delegation tool were used as teaching tools in the VET sector to describe the role and scope of the AHA, while AHLs, AHPs and AHAs reported use of these two resources and the clinician checklist to prepare the workplace for an AHA. AHL participants from the aged care sector reported using the position description and delegation tool resources to inform shaping of the AHA role and clarify scope of practice to attract the right candidate in the stages of career preparation and development.

### Tier 2 – Career development

AHA participants from the health and disability sectors discussed factors influencing their careers and the ways in which they had used the CPD log and clinician checklist to advance their career. In both the health and disability sectors, the position description was used by AHLs to enable professionalism, consistent expectations and identification of desired soft skills and capabilities at point of recruitment in the career development phase, while the clinician checklist was used to set the relationship between AHPs and AHAs. Health sector AHA participants reported utilising the CPD log and the position description resource to support development and recognition of skills, scope of practice and progression to higher grades. For AHPs in the health sector, the position description and clinician checklist provided consistent expectations of AHA roles, and AHLs referenced the position description to support clarity of scope of practice for the AHA role and performance management.

### Tier 3 – Career trajectory

Indicative of a more established AHA workforce in the health sector, compared with disability, followed by the aged care sector, participants from the health sector reported utilisation of the resources across all three career pathway tiers. AHPs and AHLs were predominantly represented in the use of the position description and clinician checklist in the career trajectory tier of the model. While AHAs did not reference utilising the two tools at career trajectory stage, AHPs in health reported using the position description to assist in understanding the stretch goals for an AHA to move between grade two and grade three roles, and in turn the difference in delegation practice required. AHLs in disability and health, utilised the position descriptions for internal recruitment of more senior AHAs, and the clinician checklist to further explore delegation practice amongst experienced AHP an AHA staff.

## Discussion

The findings of this study suggest that the co-production of resources with a framework, or recommendation for use, may reduce barriers to uptake, which were previously noted for frameworks in this field, that were not associated with relevant and readily-accessible resources. This study provides new insights about the resources that are deemed useful in optimising the development and utilisation of AHA workforces in a Victorian context, across the health, disability and aged care sectors. Role, sector, location and size of workplace influenced adoption of resources, even when appetite was comparable. Participants from smaller organisations reported readily adopting the resources as they were, with only minor local adaptations. AHAs participants reported using the resources to consider how to develop and grow in their career while AHL and the VET sector participants were more likely to use the resources to prepare an AHA for the workforce. AHPs reported utilising the resources to increase their awareness and knowledge of the role and scope of an AHA, and to support of the growth and development of the AHAs they supervised. Resources were reportedly used in elements of career preparation, development, and trajectory, across sectors in varying degrees. This was dependent on workplace readiness for change, informed by the factors of existing AHA workforce, size and location of workplace, and sector and funding stipulations.

Consistent with prior studies, both national and international, participants expressed common barriers to optimal AHA utilisation: the AHP perceived threat of the AHA role [14]; limited use of existing guidance frameworks without explicit resources [3, 15]; inconsistent pre-employment training [3]; a lack of clarity in scope of practice and role [4, 6, 16, 17]; a lack of professional development opportunities targeted to AHAs [4, 6]; AHA career stagnation [3, 15]; a general lack of empowerment on the part of the AHA role [4, 18–21], and differing resource need dependent on geographical location [5, 22, 23]. Where many participants reported cross-reference or direct adoption, this study supports the use of consistent and readily accessible resources to address these common barriers.

The appetite for developing and optimally utilising AHA workforce was clear across roles, sector and geographical location with some resources being used throughout the career pathway (Fig. 3) for this purpose. At no point was a perceived threat of the AHA role expressed by study participants.

Participants reported accessing the suite of online resources, predominantly to cross-reference current documentation, in order to introduce AHA positions in their local context. This engagement is encouraging since Huglin et al. (2021) [3] identified sub-optimal implementation of existing frameworks such as the Supervision and Delegation and workforce planning frameworks [1] and Victorian Assistant Workforce Model [24] as one barrier to optimal AHA utilisation. In this study, participants across sectors, inclusive of AHAs, and AHPs, referred to the ready-to-use nature of the resources and the ease of adopting them with minor amendments. In environments where resources for development of new workforce tools are often scarce, this may indicate that practical and ready-to-use resources may reduce barriers to optimal development and utilisation of the AHA workforce. However, utilisation of the resources appeared to differ, dependent on participant role, sector and geographical location. VET sector participants reported that these resources, when used as education aides, may assist in offering consistent messaging to learners as to the role of an AHA and the likely employment opportunities post training, and identify those suited to the course. While it is agreed that the resources have the potential to address inconsistencies in AHA pre-employment training, VET sector participants reported feeling limited by compliance requirements.

Clarity of scope and role of the AHA has long been purported as a barrier to optimal utilisation [4, 6] and as identified by Sarigiovannis and colleagues (2022) [16] it is often the individual AHP and AHA relationship that influences delegation practice in the absence of clarity of scope and role. Participants from the disability sector in our study, both AHAs and AHPs, reported downloading resources to clarify scope. This need for more clarity is likely attributed to lack of detail in funding guidelines about expected AHA scope of practice or minimum qualifications. This study identified across sector and participant groups, that individuals, who downloaded the resources, sought to clarify AHA scope of practice and role by doing so, potentially resolving this barrier to optimal utilisation.

Lack of career progression has been cited as a barrier to retention of AHAs for some time [6, 15] with previous studies reporting that AHAs can experience career stagnation and a desire for career opportunities that matched their interests and skills [4, 6]. Participants in this study reported utilising the learning needs identification, position descriptions and CPD log resources to address this barrier.

When we consider participants’ use of the learning needs identification and CPD log, this study’s findings suggest that to advance the ongoing development and optimal utilisation of this workforce, strategies may be needed, to empower AHAs to recognise and communicate their learning needs. King et al. (2022) [4] identified the lack of AHA voice in previous studies of AHA utilisation and that this in itself may be an obstacle to truly understanding the barriers to utilisation. This study extends the findings published by King and colleagues (2022) [4] by including the perspectives of AHAs, along with AHPs and AHLs, all of whom had slightly different perceptions of why and how the resources may support optimal utilisation of the AHA workforce. Empowerment of assistant workforces in various settings is also reported as a factor in retention, job satisfaction and high-quality service delivery [18, 21] and this phenomenon is not unique to the care sector. The hospitality industry also recognises the importance of empowering employees [19]. Specifically, AHAs have reported that a lack of targeted professional development is a barrier to their optimal utilisation [4, 6, 15]. Participants in this study indicated the learning needs identification and continuing professional development resources allowed for empowerment of the AHA, as leader of their own development.

Studies in Western Australia [23] and Queensland [5] have identified differences in the needs of remote and regional settings, compared to metropolitan settings, when it comes to workforce development initiatives. In this study, minimal variation between Victorian regional and metropolitan participants was noted, as potentially some of the common barriers to regional access to resources were eliminated by the fact that the recommendations and resources were available online in an editable and free version. Potentially due to urban spread and shared workforce shortages [2] across regional and metropolitan areas in Victoria, the two geographical areas have more in common when it comes to workforce development. However, one size does not fit all and size and sector of a workplace may influence utilisation of AHAs, as much as geographical location.

This study establishes there is an appetite for optimisation of the AHA workforce in Victoria, Australia. Further we have identified specific resources which may assist participants and workplaces to reduce the barriers to AHA workforce optimisation. Additional longitudinal studies in this field are needed to further understand what sustains, an appetite for, and application of, the resources.

### Study strengths and limitations

This study contained numerous methodological strengths, while acknowledging study limitations. The research team included AHA and AHPs with clinical, research, management and policy experience, who engaged in team reflexivity and met regularly during the project. Purposive quota sampling ensured representation of participants across key domains. The inclusion of solely Victorian participants, may limit the transferability of this study’s findings, however, the findings of this study are consistent with the work of others investigating the support workforce in other Australian and international contexts. While all sectors (healthcare, disability, aged care and VET) were represented at each stage of data collection, participants in this study were predominantly from the healthcare sector. It is possible that more participants from the alternate sectors may have introduced new information. However, post-interview debriefing and review of existing literature indicated that this was unlikely.

It is to be noted that not all resources marked as intended to be downloaded were actually downloaded, in some instances this number was less. However, the overall pattern of downloads and those discussed in the semi-structured interviews remain comparable (Additional file 3).

Interviewers were not in a position to influence the selection of the resource that was discussed during the interview. This was reliant on the participant having had the opportunity to review or use the resource. The semi-structured interview resource discussions numbers are not reflective of the popularity of a particular resource when considering overall resource downloads, however by allowing participants to self-select which resources to discuss the results are reflective of the resources that resonated most with participants. Finally, the lower proportion of data about how the resources were being used, is likely attributed to constrained time to use resources before being interviewed (less than a fortnight between download and interview in some instances).

## Conclusions

Study participants, from all sectors, downloaded the ready-to-use resources that were on offer to support optimal development and utilisation of the AHA workforce in these sectors and address well documented barriers to AHA workforce optimisation. Having ready-to-use, accessible implementation resources associated with frameworks and recommendations, assist all relevant sectors in part, on their journey to optimal AHA workforce development and utilisation. The findings of this study are based on the views of key participants from predominantly the health sector in Victoria, Australia. Further investigation is required to explore the transferability of these findings to all sectors employing AHAs in national and global contexts.

### Electronic supplementary material

Below is the link to the electronic supplementary material.

Additional File 1. List of Resources released online.

Additional File 2. Heat map of potential interview participants.

Additional File 3. Representation of intended downloads, actual downloads and interview participant selection of resources for discussion.

Additional File 4. Semi-structured interview template.

Additional File 5. Pictorial representation of coding categories and themes within each.

Additional File 6. Codes by Career Pathway Tier.

Additional file 7. Resource intention to download between March and June as per geographical location.

## Data Availability

Data is provided within the manuscript or supplementary information files.

## Abbreviations

AHA: Allied health assistant
AHP: Allied health professional
COREQ: Consolidated Criteria for Reporting of Qualitative Research
VET: Vocational education and training
VAWM: Victorian Assistant Workforce Model
NDIS: National Disability Insurance Scheme
RTO: Registered Training Organisation
