# Supplementary material for: The evaluation of health, disability and aged care-sector engagement with resources designed to support optimisation of the allied health assistant workforce: a qualitative study
Source: BMC Health Serv Res. 2024 Jul 26;24:848. doi: 10.1186/s12913-024-11253-z (PMC11282609; doi:10.1186/s12913-024-11253-z)
Supplement: Supplementary file 1 — Additional File 1. List of Resources released online. [file 12913_2024_11253_MOESM1_ESM.pdf]

Additional file 1. List of Resources released online.

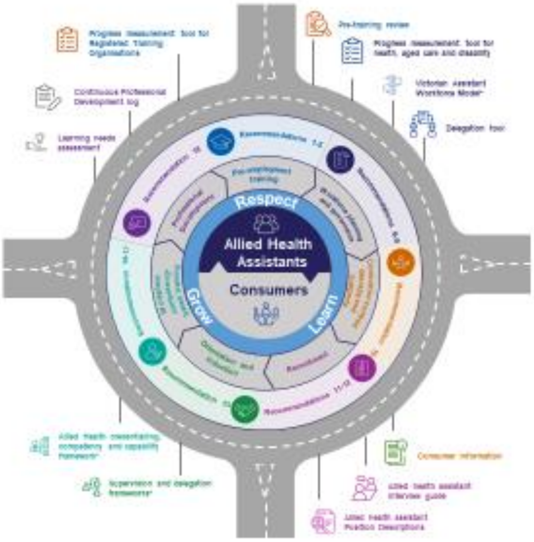

Progress measurement tool (for health, aged care, and disability)
Progress measurement tool (for Registered training Organisations)
Clinician checklist (for Allied health professionals and Allied health assistants)
Consumer information – 'Allied health assistants and you'
Consumer information – 'Allied health assistants and you (Easy English)'
Registered Training Organisation pre-training review
Position Description – Grade 1 Allied health assistant
Position Description – Grade 2 Allied health assistant/Level 1 Therapy Assistant
Position Description – Grade 3 Allied health assistant/Level 2 Therapy Assistant
Allied health interview guide
Allied health assistant learning needs
Allied health assistant Continuing Professional Development log
Allied health assistant delegation tool
Business case

The full resources can be found here: Victorian allied health assistant workforce recommendations and resources | health.vic.gov.au
