# Supplementary material for: The evaluation of health, disability and aged care-sector engagement with resources designed to support optimisation of the allied health assistant workforce: a qualitative study
Source: BMC Health Serv Res. 2024 Jul 26;24:848. doi: 10.1186/s12913-024-11253-z (PMC11282609; doi:10.1186/s12913-024-11253-z)
Supplement: Supplementary file 2 — Additional File 2. Heat map of potential interview participants. [file 12913_2024_11253_MOESM2_ESM.pdf]

Additional file 2. Heat map of potential interview participants.

Stakeholder /Sector	*Disability*	*Health*	*VET*	*Other*	*Aged Care*	*Withdrew*	*Grand Total*
AHA	6	54		2	2		64
AH Leader	24	33		7	5		69
AHP	14	17	1	4	1		37
VET	1	2	13	2			18
Other		1					1
Withdrew							1
Grand Total	**45**	**107**	**14**	**15**	**8**	**1**	**190**
