# Supplementary material for: The evaluation of health, disability and aged care-sector engagement with resources designed to support optimisation of the allied health assistant workforce: a qualitative study
Source: BMC Health Serv Res. 2024 Jul 26;24:848. doi: 10.1186/s12913-024-11253-z (PMC11282609; doi:10.1186/s12913-024-11253-z)
Supplement: Supplementary file 3 — Additional File 3. Representation of intended downloads, actual downloads and interview participant selection of resources for discussion. [file 12913_2024_11253_MOESM3_ESM.pdf]

Additional file 3. Representation of intended downloads, actual downloads and interview participant selection of resources for discussion. The forty-five interviews resulted in eighty-one resources being discussed.

Resource	Total Intended downloads	Total Actual downloads	Interview candidate download and discussion					
			Health	Aged Care	Disability	VET	Other	Resource discussed at interview
**Progress measurement tool (for health, aged care, and disability)**	308	133	2	1	1			4
**Progress measurement tool (for Registered training Organisations)**	146	133	1			4		5
**Clinician checklist (for Allied health professionals and Allied health assistants)**	361	217	6	2	3		1	12
**Consumer information – 'Allied health assistants and you'**	299	157	1	2	1	1	1	6
**Consumer information – 'Allied health assistants and you (Easy English)'**	210	126						
**Registered Training Organisation pre-training review**	150	102	1			4		5
**Position Description – Grade 1 Allied health assistant**	0	0						
**Position Description – Grade 2 Allied health assistant/Level 1 Therapy Assistant**	291	150	3	1	7	4		16
**Position Description – Grade 3 Allied health assistant/Level 2 Therapy Assistant**	321	189						
**Allied health interview guide**	297	160	4	1				5
**Allied health assistant learning needs**	355	176	3		2		1	6
**Allied health assistant Continuing Professional Development log**	337	150	3		2			5
**Allied health assistant delegation tool**	353	216	7	4	3	1	2	17
**Business case**	0	0					1	1
**Total**	3,428	1909	31	11	19	14	6	81
