# Supplementary material for: The evaluation of health, disability and aged care-sector engagement with resources designed to support optimisation of the allied health assistant workforce: a qualitative study
Source: BMC Health Serv Res. 2024 Jul 26;24:848. doi: 10.1186/s12913-024-11253-z (PMC11282609; doi:10.1186/s12913-024-11253-z)
Supplement: Supplementary file 4 — Additional File 4. Semi-structured interview template. [file 12913_2024_11253_MOESM4_ESM.pdf]

Additional file 4. Semi-structured Interview template

Interview background and purpose

The purpose of this interview is to explore the current use of the implementation resources within the Victorian AHA Workforce Recommendations.

The resources have been developed to identify opportunities to improve the utilisation of the Allied Health assistant workforce in the Victorian health, aged care and disability sectors.

The areas to be covered in this interview are:

- the appetite for the implementation resources in each sector;
- the intended use of the resources in each sector;
- the actual use and usability of the resources in each sector
- the expected impact of each tool on AHA utilisation in each sector;
- the actual impact of each tool on AHA utilisation in each sector;
- what further resources would be useful in achieving optimal utilisation of AHAs in each setting.

The priority ordering of discussion topics for the semi-structured interview will be:

Appetite

1. When you downloaded [insert resource title], what was your purpose? (i.e. what did you expect from the tool? What led you to download the tool?)
    a. Are you applying the resource with an existing AHA workforce?
    b. Are you considering employing AHAs? (if don't have an existing workforce)
    c. Do you already have a similar resource and using this to compare?
    d. Is there any other reason why you've downloaded the resource?

Usability

2. Did [insert resource title] assist in meeting your intended purpose?
    a. If so, how?
    b. If not, what had you expected/required that the resource did not offer?
    c. Did the resource require adaptation for your setting?
    d. Have you continued to use the resource?

Change in practice

3. Has the [insert resource title] assisted in increasing utilisation of AHAs in your setting through:
    a. Increased delegation
    b. Increased recruitment
    c. Increased planning
    d. Increased uptake

Sustainability

4. Do you think you will continue to use the [insert resource title]?
    a. If not, why not?
    b. If so, will it require further adaptation?

Extendibility

5. Do you see this [insert resource title] resource being useful to implementing /defining other support workforces relevant to your setting?
6. What other resources would be useful in your setting to increase utilisation/uptake of AHAs?
7. Compared to the resource that we have discussed in depth are there any other resources that you used or will continue to use? What did you think of it?

Wrap up

8. Ascertain if anyone wants to add anything else to the discussion.
9. Ascertain if any questions for the facilitator.

Semi-structured interview script

Welcome and initial set up

Hello ________________________

Thank you for taking the time to participate in our Victorian allied health assistant workforce research project.

My name is ________________ and I work as___________________. I will be interviewing you today. I would also like to introduce you to ____________________, who will be the note taker for the interview.

As you are aware you have provided written consent to participate. To maintain confidentiality your name will not be noted as part of the transcription of this interview. This is planned to run for up to 40 minutes. A recording of the session will now start and if you feel uncomfortable during the interview, just raise your hand and I can stop the recording (start the recording).

Introduction to the study and interview ground rules

As you know, this interview is being audio taped and it will be transcribed verbatim. Your identity will be protected in the transcript and I will use your coded participant number on the tape.

I want you to speak freely during this session. There's no right or wrong answer and anything that you do say will remain confidential. It is important to note though, that it will only remain confidential if you keep the information confidential to yourself as well. So even though the transcript won't go anywhere, if you talk outside the room about what we say then that breaks the confidentiality, so you need to keep that in mind.

If you do feel uncomfortable during the interview, just raise your hand and I can stop the recording. You are free to leave at any time, and if you do decide to leave, your participation will stop there. However, as I can't undo what's already on the tape, your contribution up until you leave cannot be removed.

I want to explore the opportunities to adapt or improve the resources provided for increasing utilisation of the allied health assistant workforce in the Victorian health, aged care and disability sectors. AHAs are an important part of increasing the ability of the allied health workforce to better meet the care needs of older adults, people with a disability and people with health conditions such as chronic diseases. The research will be used to continue to inform recommendations and implementation resources for the Victorian AHA workforce on how to best optimise the use of AHAs in the Victorian health, aged care and disability sectors.

We have already gained some preliminary results from local testing of the resources with the Project Steering Committee, which is made up of stakeholders from the Victorian health, aged care, and disability and VET sectors, and a workforce survey.

I would like to hear your views on the implementation resources you have recently downloaded. I would also like to hear what resources your workplace currently uses to support the AHA workforce, for example, a competency framework or the supervision and delegation framework for AHAs, and how they are applied in practice (applicability, ease of use, support required to meaningfully use the tool). Lastly, I am also interested in as to other resources you feel are needed to better utilise AHAs in your setting.

I would like you to introduce yourself by professional background [facilitator introduces herself as an example – Hi, I am a physiotherapist with 20 years' experience in hospital and community rehab [similar introduction by all interviewees]

Have you participated in a semi-structured interview before? I am using an interview to capture your experience of the AHA workforce implementation resources and your in-depth thoughts and ideas to how to promote utilisation of AHAs. There are no right or wrong answers, rather the intention is to capture your thoughts and opinions. I want you all to feel comfortable sharing, so everything that is said in the room stays here.

Are there any questions before we start? Let us start

Semi-structured interview questions

Appetite

We have learnt from the results of our broad consultations with the Steering Committee that there is a great need for the recommendations we have proposed however they are difficult to execute without the implementation resources to do so.

1. When you downloaded [insert resource title] resource, what was your purpose? (i.e. what did you expect from the tool? What led you to download the tool?)
    a. Are you applying the tool with an existing AHA workforce?
    b. Are you considering employing AHAs? (if don't have an existing workforce)
    c. Do you already have a similar tool and using this to compare?
    d. Is there any other reason why you've downloaded the resource?

Usability

The implementation resources have been designed to allow a workplace to put in action the relevant recommendation.

2. Did [insert resource title] assist in meeting your intended purpose?
    a. If so, how?
    b. If not, what had you expected/required that the resource did not offer?
    c. Did the resource require adaptation for your setting?
    d. Have you continued to use the resource?

Change in practice

The aim of the implementation resources based on the consultation that they are designed on, is to assist workplace settings to achieve an increased utilisation of Allied Health Assistants.

3. Has the [insert resource title] resource assisted in increasing utilisation of AHAs in your setting resulted in?
    a. Increased delegation

b. Increased recruitment
c. Increased planning
d. Increased uptake

Sustainability

The implementation resources have been designed to allow for the growth of the AHA workforce and the extension of the AHA workforce into settings where they may not have traditionally been used.

4. Do you think you will continue to use the [insert resource title] resource?
    a. If not, why not?
    b. If so, will it require further adaptation?

Extendability

The Allied health assistant workforce have had a large amount of thought and process put into place over the last 15 years by the department of health. However, there are many other support workforces working alongside Allied health assistants who lack the same level of definition. The implementation resources have been designed to allow for the growth of the AHA workforce.

5. Do you see this [insert resource title] resource being useful to implementing /defining other support workforces relevant to your setting?
6. What other resources would be useful in your setting to increase utilisation/uptake of AHAs?

Wrap up

7. Ascertain if anyone wants to add anything else to the discussion.
8. Ascertain if any questions for the facilitator.

Thank you for your participation. The next steps in the study are to analyse and theme the findings, and then use this information to inform the final Victorian AHA workforce recommendations and implementation resources.

Sample probes to obtain richer responses

- “Please tell me more about that”
- “Could you please explain what you mean by”
- “Can you tell me something about”
