# Supplementary material for: The evaluation of health, disability and aged care-sector engagement with resources designed to support optimisation of the allied health assistant workforce: a qualitative study
Source: BMC Health Serv Res. 2024 Jul 26;24:848. doi: 10.1186/s12913-024-11253-z (PMC11282609; doi:10.1186/s12913-024-11253-z)

Additional file 5. Pictorial representation of coding categories and themes within each.

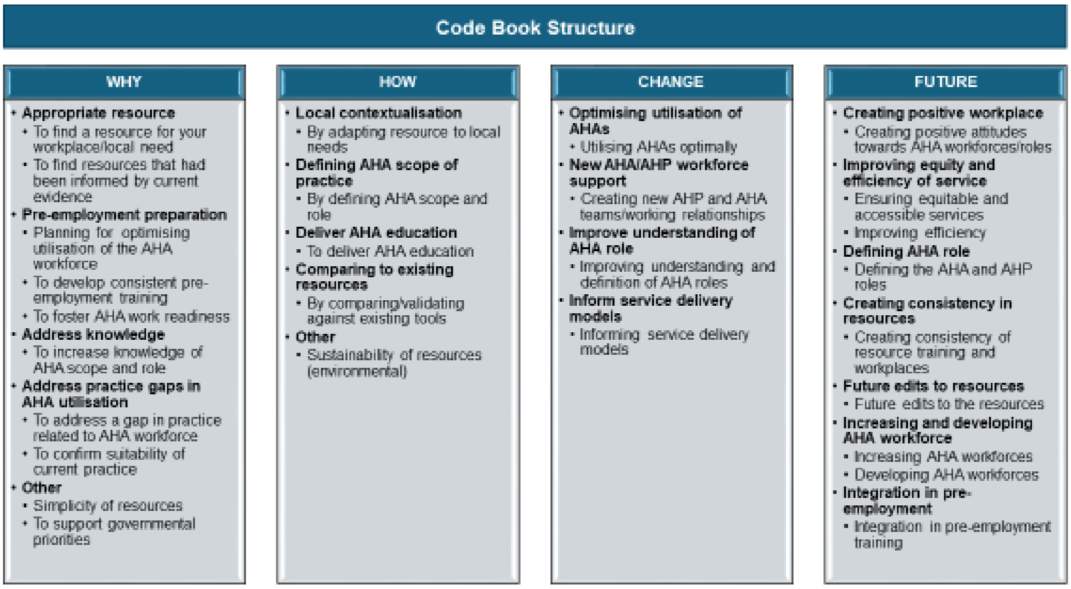

Supplement: Supplementary file 5 — Additional File 5. Pictorial representation of coding categories and themes within each. [file 12913_2024_11253_MOESM5_ESM.pdf]
