# Supplementary material for: The evaluation of health, disability and aged care-sector engagement with resources designed to support optimisation of the allied health assistant workforce: a qualitative study
Source: BMC Health Serv Res. 2024 Jul 26;24:848. doi: 10.1186/s12913-024-11253-z (PMC11282609; doi:10.1186/s12913-024-11253-z)
Supplement: Supplementary file 6 — Additional File 6. Codes by Career Pathway Tier. [file 12913_2024_11253_MOESM6_ESM.pdf]

Additional file 6. Codes by Career Pathway Tier

Career Pathway Tier

Preparation

- **Appropriate resource**
  - To find a resource for your workplace/local need
  - To find resources that had been informed by current evidence
- **Pre-employment preparation**
  - Planning for optimising utilisation of the AHA workforce
  - To develop consistent pre-employment training
  - To foster AHA work readiness
- **Address knowledge**
  - To increase knowledge of AHA scope and role
- **Local contextualisation**
  - By adapting resource to local needs
- **Defining AHA scope of practice**
  - By defining AHA scope and role
- **Comparing to existing resources**
  - By comparing/validating against existing tools
  - **Improve understanding of AHA role**
    - Improving understanding and definition of AHA roles
- **Integration in pre-employment**
  - Integration in pre-employment training
- **Creating consistency in resources**
  - Creating consistency of resource training and workplaces
- **Defining AHA role**
  - Defining the AHA and AHP roles

Development

- **Deliver AHA education**
  - To deliver AHA education
- **Other**
  - Sustainability of resources (environmental)
- **New AHA/AHP workforce support**
  - Creating new AHP and AHA teams/working relationships
- **Future edits to resources**
  - Future edits to the resources
- **Creating positive workplace**
  - Creating positive attitudes towards AHA workforces/roles

Trajectory

- **Optimising utilisation of AHAs**
  - Utilising AHAs optimally
- **Inform service delivery models**
  - Informing service delivery models
- **Address practice gaps in AHA utilisation**
  - To address a gap in practice related to AHA workforce
  - To confirm suitability of current practice
- **Other**
  - Simplicity of resources
  - To support governmental priorities
- **Increasing and developing AHA workforce**
  - Increasing AHA workforces
  - Developing AHA workforces
- **Improving equity and efficiency of service**
  - Ensuring equitable and accessible services
  - Improving efficiency
