# Supplementary material for: The evaluation of health, disability and aged care-sector engagement with resources designed to support optimisation of the allied health assistant workforce: a qualitative study
Source: BMC Health Serv Res. 2024 Jul 26;24:848. doi: 10.1186/s12913-024-11253-z (PMC11282609; doi:10.1186/s12913-024-11253-z)
Supplement: Supplementary file 7 — Additional file 7. Resource intention to download between March and June as per geographical location. [file 12913_2024_11253_MOESM7_ESM.pdf]

Additional file 7. Resource intention to download between March and June as per geographical location

Metropolitan.

Metropolitan	*1st Downloaded*	*2nd Downloaded*	*3rd Downloaded*	*4th Downloaded*
Health	Delegation tool (142)	Clinician checklist (142)	Learning needs (141)	Interview guide (136)
Disability	Delegation tool (16)	Clinician checklist (15)	Learning needs (15)	Progress measurement tool (health, aged, disability) (15)
Aged Care	Grade 3 PD (10)	Clinician checklist (8)	Grade 2 PD (8)	Progress measurement tool (health, aged, disability) (8)
VET	Progress measurement tool (RTO) (12)	Interview guide (12)	Learning needs (12)	Delegation tool (11)
Other	Clinician checklist (8)	Delegation tool (7)	Learning needs (7)	Interview guide (6)
Metropolitan Total	Clinician checklist (184)	Delegation tool (182)	Learning needs (181)	CPD (171)

Regional

Regional	*1st Downloaded*	*2nd Downloaded*	*3rd Downloaded*	*4th Downloaded*
Health	Learning needs (76)	CPD (76)	Clinician checklist (75)	Delegation tool (69)
Disability	CPD (20)	Clinician checklist (20)	Consumer resource (20)	Learning needs (19)
Aged Care	Delegation tool (1)	Clinician checklist (1)	CPD (1)	Learning needs (1)
VET	Delegation tool (3)	Clinician checklist (3)	CPD (3)	Learning needs (3)
Other	Delegation tool (4)	Clinician checklist (4)	CPD (4)	Progress measurement tool (health, aged, disability) (4)
Regional Total	Learning needs (104)	CPD (104)	Clinician checklist (103)	Delegation tool (95)
